# Effect of Street Performance (Busking) on the Environmental Perception of Public Space

**DOI:** 10.3389/fpsyg.2021.647863

**Published:** 2021-03-30

**Authors:** Robbie Ho, Wing Tung Au

**Affiliations:** ^1^Division of Social Sciences, Humanities and Design, College of Professional and Continuing Education, The Hong Kong Polytechnic University, Hung Hom, Hong Kong; ^2^Department of Psychology, The Chinese University of Hong Kong, Shatin, Hong Kong

**Keywords:** street performance, street music, busking, environmental perception, public space, public place

## Abstract

This is the first experimental study testing the effect of street performance (aka busking) on the subjective environmental perception of public space. It is generally believed that street performance can enhance people’s experience of public space, but studies advocating such a view have not used a control group to explicitly verify the effect of street performance. In response to this methodological limitation, we conducted two studies using experimental design. Study 1 (*N* = 748) was an online computer-based study where research participants evaluated the extent to which the presence vs. absence of street performance could change their perception of public space. Study 2 (*N* = 162) was a between-group quasi-experiment in an actual public space where people physically present in the space evaluated the perception of the space with vs. without street performance. Overall, we found converging results that street performance could make public space more visitable, more restorative, and more preferable. The current findings not only fill in a gap in the literature on street performance, but they also inform the policy making and regulations of street performance.

## Introduction

### Street Performance

Street performance, or busking, refers to the act of performing or entertaining in a public space with the intention of seeking voluntary donations from passersby. Street performers, or buskers, are people who conduct such an act. Street performance has a long history; wandering minstrels, troubadours, mountebanks, comedians, and showmen in street fairs performed on the street to earn a living (Boyle, 1978; Cohen and Greenwood, 1981). This tradition extends into the modern day. Today it is still common to encounter street performance in public spaces where we commute and conduct our everyday activities. Street performance is spontaneous and takes place ephemerally often in transitional places such as transit stops, streets, and squares or plazas (D. Smith, 2011). Bouissac (1992) describes street performance as “the modern, urban version of an ancient mode of economic survival” (p. 14). Donation to street performance is typically voluntary. Spectators of street performance freely decide whether to donate money or not, and if they do they freely decide how much to donate. Thus, street performance can be considered as a public good that is open to freeriding (Kushner and Brooks, 2000). Many street performers display signs and/or receptacles such as hats and instrument cases as a prompt for donation (T. Smith, 2016). Street performance comes in various forms, although a general differentiation can be made between musical and non-musical (e.g., juggling, miming, dancing, and magic, etc.) busking (Boyle, 1978; Campbell, 1981; Harrison-Pepper, 1990). Musical busking is a major form of street performance across cultures and through history (Boyle, 1978; Campbell, 1981; Cohen and Greenwood, 1981; Harrison-Pepper, 1990; Tanenbaum, 1995; Simpson, 2011; Doughty and Lagerqvist, 2016). This paper focuses on musical busking as one of the representations of street performance.

### Public Space

Public space is a broad concept and refers to places that are open to all and allow a wide range of activities to take place. Carr et al. (1992) define public spaces as “open, publicly accessible places where people go for group or individual activities… Some are under public ownership and management, whereas others are privately owned but open to the public” (p. 50). According to Project for Public Spaces (2018), public spaces are often “used by many different people for many different purposes at many different times of the day and the year” (p. 1). Thus, public spaces have no single purpose and serve no single person. Nonetheless, public spaces can be categorized according to their specific functions and purposes (Carmona, 2010). In studying the environmental perception of public space, Ho and Au (2020) reviewed several typologies of public space evolved from previous studies (Carr et al., 1992; Gehl and Gemzøe, 2001; Stanley et al., 2012) and identified 12 major types of public space: transport facility, street, square, recreational space, found neighborhood space, park, memorial, market, playground, community open space, indoor marketplace, and waterfront – each serves a different function or purpose. Table 1 presents these 12 types of public space and their definitions. This typology will serve to operationalize public space throughout this paper.

**TABLE 1:** 12 Major types of public space.

**Space type**	**Definition**
Transport facility	Public space for transport facilities such as transit stations or stops for subways or buses
Street	Pedestrian and vehicular corridor where people move on foot
Square	Multifunctional space available to all people
Recreational space	Specialized space designed or used for sports or exercises
Found neighborhood space	Vacant or undeveloped space that is either ignored or not intended for a specific use
Park	Green area intended for social activities
Memorial	Space that memorializes people or important events
Market	Outdoor or exterior space used for shopping
Playground	Play area that includes play equipment (e.g., slides and swings)
Community open space	Space designed, developed, or managed by local residents on vacant land
Indoor marketplace	Indoor shopping area
Waterfront	Open space along waterways in cities

### Effect of Street Performance on the Environmental Perception of Public Space

We take an experimental approach to test the effect of street performance on the subjective environmental perception of public space. Literature regarding street performance has concentrated on the history of street performance (Campbell, 1981; Cohen and Greenwood, 1981; M. Smith, 1996), case studies of the street performances in specific locations (Prato, 1984; Harrison-Pepper, 1990; Tanenbaum, 1995; Marina, 2018), and life stories of street performers (Moore, 1974; Press and McNamara, 1975; Condos, 1976; Palmquist, 1984; Gomes, 2000; Rebeiro Gruhl, 2017). Others have studied street performance from the economic (Kushner and Brooks, 2000), legal and legislative (McNamara and Quilter, 2016; Juricich, 2017), urban design and policy (Astor, 2019; Clua et al., 2020), and spectator experience (Ho and Au, 2018; Ho et al., 2020) perspectives. Last but not least, there is the discourse that street performance can enhance people’s experience of public space (Simpson, 2011; Doughty and Lagerqvist, 2016; Doubleday, 2018), and that is of interest to the current paper.

Music is a common experience. Even if not from street performance, we often encounter music in public space. An obvious case is the background music in service environments such as supermarket, shopping mall, and restaurant, etc. Background music in public settings as such can impact our perception of the settings. Numerous studies have demonstrated that shoppers of supermarket and retail mall perceive the store and sales personnel more favorably and spend more time and more money in the store if they like the background music being played (Herrington and Capella, 1996; Dubé and Morin, 2001; Vida et al., 2007; Andersson et al., 2012; Yi and Kang, 2019). Effect of music seems to also apply in restaurant. Compared to patrons in restaurant with no music, patrons in restaurant with music – no matter classical, jazz, or pop – are willing to spend a greater amount of money on their meals (Wilson, 2003). Others have found that songs with prosocial lyrics in particular can increase restaurant patrons’ tipping behavior (Jacob et al., 2010). These findings clearly show that music in everyday situation can affect how people perceive and interact with the immediate environment. Thus, we should also expect that street performance can impact our perception of public space.

There is some research support for the view that street performance can enhance people’s experience of public space. Majority of the advocates based the conclusion on observation. Whyte (1980, 1988) observed in New York that street performance could make public squares more amicable. Tanenbaum (1995) found that train riders felt safer with street music being present around New York subway stations. In Bath, United Kingdom, Simpson (2011) observed that street performance could enhance the sociability and conviviality of public space. In Stockholm, Doughty and Lagerqvist (2016) found that people perceived a public square as friendlier when street music was present. There are also survey findings that revealed the potential benefits of street performance. In Hong Kong, people reported positive feelings toward the street environment surrounding a street performance (e.g., “*This performance made me love this place.*” and “*This performance made me feel I belonged to this place.*”; Ho and Au, 2018; Ho et al., 2020). In Santa Monica, shoppers of a shopping promenade thought that street performance was important to the attraction of the area (Doubleday, 2018). Overall, these findings support the view that street performance is associated with a positive perception of public space. But there is a methodological limitation that none of the abovementioned studies have used a control group – i.e., a setting without street performance – as a benchmark to explicitly verify the effect of street performance. This paper seeks to fill in the research gap by revisiting the effect of street performance on the perception of public space with an experimental approach.

### Research Hypotheses: Visitability, Restorativeness, and Preference

We examine the effect of street performance on the perceived visitability, restorativeness, and preference of public space. Previous studies have established the general view that people perceive public space with street performance as amicable and sociable (Whyte, 1980, 1988; Tanenbaum, 1995; Simpson, 2011; Doughty and Lagerqvist, 2016). We will examine this effect in terms of visitability, which refers to the extent to which a given environmental setting is perceived as friendly and worth visiting and spending time about. This notion of visitability was first coined by Abdulkarim and Nasar (2014a; 2014b). We draw on their operationalization and hypothesize that:

The presence of street performance will increase the visitability of public space. (H1)

We will also examine the effect of street performance in terms of restorativeness, which refers to the extent to which a given environmental setting allows its viewers to relax and have a sense of temporary escape from daily stressors. The notion of restorativeness originated from Attention Restoration Theory (Kaplan and Kaplan, 1989; Kaplan, 1995), which suggests that restorative environments are preferable as they allow people’s attentional system to relax and recover. According to the theory, an environment is considered as restorative if it is interesting enough for people to want to engage with it, allows its viewers to feel immersed, and requires effortless attention from its viewers. Previous research has looked at how the restorativeness of public space could be enhanced by the presence of trees and street vegetation (Lindal and Hartig, 2015; Rašković and Decker, 2015). In this paper we apply the theory to the context of street performance. Street performance should enhance the restorativeness of public space because it offers an interesting and immersive experience that captures people’s attention effortlessly. Hence, we hypothesize that:

The presence of street performance will increase the restorativeness of public space. (H2)

Finally, we will examine the effect of street performance in terms of people’s overall preference of public space. Overall preference, or simply preference, refers to the overall liking of public space. Preference is a common notion in studies related to public space (Herzog, 1992; Herzog and Gale, 1996; Herzog and Leverich, 2003; Herzog and Bryce, 2007). As discussed earlier, past studies revealed that the presence of street performance was associated with an attractive surrounding environment (Doubleday, 2018; Ho and Au, 2018; Ho et al., 2020). Hence, we hypothesize that:

The presence of street performance will increase the preference of public space. (H3)

### Practical Implication

There is a practical reason for examining the effect of street performance on the perception of public space. Despite its historical reputation and cultural charms, currently there is no global consensus on the legality of street performance (Doumpa and Broad, 2017). Busking is legal in one place but illegal in another. For instance, in Australian cities such as Sydney and Melbourne, buskers may obtain licenses for performing legally in public space with the right to accept donations (McNamara and Quilter, 2016), whereas in Hong Kong, buskers may be arrested for conducting “unauthorized charitable behavior” in public space (Lai and Da Roza, 2018). In determining the public policy and legality of street performance, one important consideration is the impact of street performance on people’s experience of public space. If street performance can enhance people’s experience of public space, then it is sensible to promote street performance through public policy and constructive regulation terms. But if street performance evidently undermines the quality of public space, then it makes sense to impose more restrictive terms to minimize its negative impact. This paper will take an experimental approach to investigate the effect of street performance on the perception of public space. Not only will our findings fill in a research gap, but they will also provide clear evidence to inform the policy making and regulations of street performance.

### Present Study

The present study takes an experimental approach to test the effect of street performance on the subjective environmental perception of public space. We hypothesize that the presence of street performance will increase the perceived visitability (H1), restorativeness (H2), and preference (H3) of public space. We conducted two studies to test these hypotheses, ethical approval was obtained prior to both studies.

Study 1 was an online, computer-based study. Research participants were recruited online and were shown computer-generated images of public spaces with street performance being superimposed on the spaces gradually. Participants reported the extent to which the presence of street performance could change their perception of the public spaces.

Study 2 was a between-group, quasi-experiment. People passing through a public space in Hong Kong were intercepted and invited to report their perception of the space. We conducted, separately, a control session and an experimental session. In the control session the public space was without street performance. In the experimental session we set up a street performance in the space. We compared the perception of the two groups to determine if the presence of street performance caused a difference.

## Study 1: Online Computer-Based Study of the Effect of Street Performance

### Study 1 Method

#### Pictorial Stimuli of Public Spaces With and Without Street Performance

**FIGURE 1:**
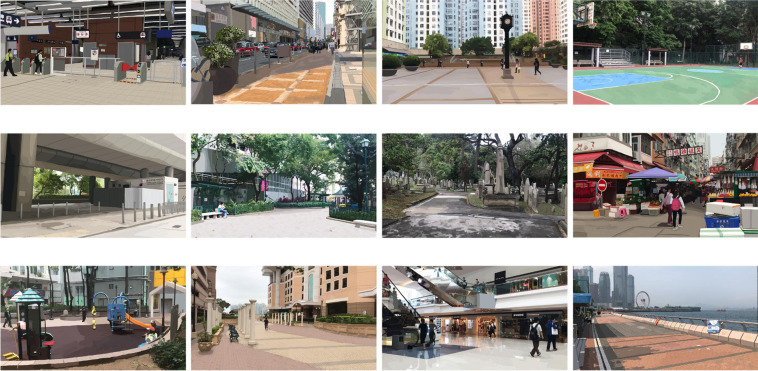
Pictorial stimuli of public spaces without street performance (top row, from left to right, transport facility, street, square, and recreational space; middle row, from left to right, found neighborhood space, park, memorial, and market; and bottom row, from left to right, playground, community open space, indoor marketplace, and waterfront).

**FIGURE 2:**
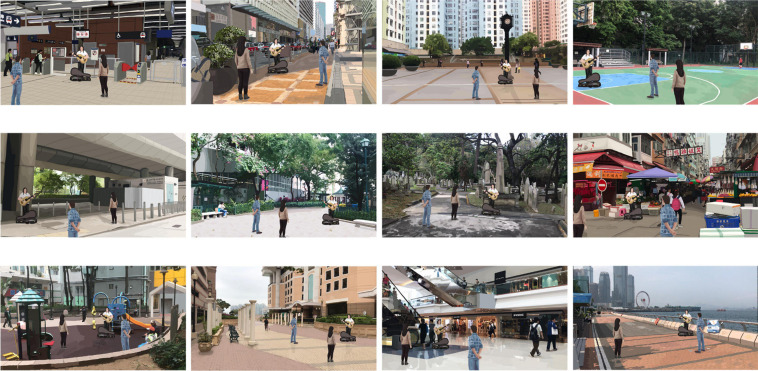
Pictorial stimuli of public spaces with street performance (top row, from left to right, transport facility, street, square, and recreational space; middle row, from left to right, found neighborhood space, park, memorial, and market; and bottom row, from left to right, playground, community open space, indoor marketplace, and waterfront).

We employed a set of computer-generated images in the graphics interchange format (GIF) to represent the 12 major types of public space (see Table 1) with and without the presence of street performance. The complete set of GIF images is provided in Supplementary Appendix A. The use of pictorial stimuli to present environments for subjective evaluation is supported by Stamps’ (2010) meta-analysis, which found that the evaluation of environments on-site and the evaluation of environments based on static media were strongly correlated (*r* = 0.86). In studying the environmental perception of public space, Ho and Au (2020) created 12 images to represent the 12 major types of public space. We used their images as the basis for generating the GIF images of the current study. Ho and Au’s original images depicted the 12 public spaces in the natural setting, i.e., without street performance (see Figure 1). On each of the images, we superimposed an animated street performance in GIF (see Figure 2). The street performance comprised a musical busker interacting with two adult passersby. The busker was holding an acoustic guitar, with an open guitar case at the foot intended as a receptacle for donations from passersby. The two passersby were placed around the busker; they were standing and facing toward the busker; and they were neither ignoring nor endorsing the busker to convey a neutral impression. The same design was applied across the 12 images. The busker and passersby varied slightly in size to fit the setting in each image naturally. In all 12 GIF images, as the public space remained constant, the street performance would fade in gradually and then disappear in an endless loop. These images presented the 12 major types of public space both before and after street performance became present.

#### Participants

Research participants were recruited on Amazon Mechanical Turk (MTurk). MTurk is an online platform where anonymous individuals from all around the world can be recruited to perform web-based tasks including online academic survey. There is research support for using MTurk as a data source. It has been demonstrated that MTurk samples respond to experimental tasks in the social sciences in a manner consistent with other common forms of convenient samples such as college students (Berinsky et al., 2012; Casler et al., 2013). In addition, we included an attention-check mechanism regarded as instructed response items (IRIs) in the current study (see Measures below). IRIs are items to which the correct answer is obvious and unambiguous; they are widely used and acceptable in survey research (Gummer et al., 2018; Kam and Chan, 2018; Kung et al., 2018) and they should be sufficient to safeguard the data quality of the current study (Goodman et al., 2013; Kees et al., 2017).

A total of 852 individuals responded to our recruitment on MTurk. Each respondent received US$1.50 for participation. A total of 748 respondents passed the attention check and their data were included in the data analysis. The retained sample comprised 357 women and 391 men whose average age was 36.3 years (*SD* = 11.1 years; 24 preferred not to answer). Table 2 presents the sample’s demographics. About three quarters of the sample lived in North America (75.1%), about a tenth in Asia (11.9%), and smaller proportions in South America (6.7%), and Europe (3.7%). There was a fair split among those who had attained a bachelor’s degree as their highest education level (41.7%), those who had not (35.2%), and those who had attained an education level above a bachelor’s degree (22.8%). Most of the sample identified themselves as middle class (60.6%), followed by working class (31.6%).

**TABLE 2:** Demographics of Study 1 sample.

	***n***	**%**
**Location**		
North America	562	75.1
South America	50	6.7
Africa	6	0.8
Europe	28	3.7
Asia	89	11.9
Australia/Oceania	1	0.1
Prefer not to answer	12	1.6
**Education**		
Some high school	1	0.1
High school diploma or equivalent	62	8.3
Vocational training	10	1.3
Some college	119	15.9
Associate’s degree	72	9.6
Bachelor’s degree	312	41.7
Some post undergraduate work	15	2.0
Master’s degree	137	18.3
Specialist degree	4	0.5
Applied or professional doctorate degree	3	0.4
Doctorate degree	12	1.6
Prefer not to answer	1	0.1
**Class**		
Poor	29	3.9
Working class	236	31.6
Middle class	453	60.6
Affluent	21	2.8
Prefer not to answer	9	1.2

#### Procedure

Participants were randomly assigned to rate one of the 12 space types. Table 3 presents the sample sizes rating the 12 space types.

**TABLE 3:** Sample sizes in Study 1.

**Space type**	***n***	**%**
Transport facility	67	9.0
Street	69	9.2
Square	63	8.4
Recreational space	66	8.8
Found neighborhood space	56	7.5
Park	62	8.3
Memorial	61	8.2
Market	56	7.5
Playground	67	9.0
Community open space	72	9.6
Indoor marketplace	53	7.1
Waterfront	56	7.5

Participants filled out an online survey to evaluate the extent to which the presence of street performance could change their perception of the assigned public space as portrayed in the GIF image. The survey began with an introduction that explicitly stated that the study was to understand human experience of public space. After giving their informed consent, participants were shown the complete set of the 12 images of public spaces in the original setting (i.e., without street performance) to help anchor their judgment. The order of the images was randomized for every participant. After viewing the set, the standard figure of the musical busker as being used in the current study along with the definition of street performance were introduced. Next, the GIF image of the assigned public space with gradual appearance of street performance was shown. The image came with the definition of the corresponding space type to prompt the participants about the space type the image was supposed to represent. The participants were also prompted to imagine that they encountered the public space on a regular basis (“*Imagine that you use this place or commute through it on a regular basis; that is, you encounter this place for your everyday activities, e.g., walking through this place to work or school, hanging out, meeting people, and shopping, etc.*”). Then, they were asked to evaluate the extent to which the presence of street performance changed their perception of the space. They were reminded to focus on the setting of the space rather than the quality of the image, and that there were no right or wrong answers. There was no restriction to how long the participants should look at the GIF image. They were free to look at the image while they performed the evaluation.

#### Measures

Participants reported the change in their perception of the public spaces in terms of visitability, restorativeness, and preference on a 7-point scale (from *much less* to *much more* coded from −3 to 3 with the midpoint *about the same* as 0). All scale items are presented in Table 4. We adopted the visitability items from Abdulkarim and Nasar (2014b) and restorativeness items from Pals et al. (2014). Preference is a general concept that is commonly studied in environmental-psychological studies (Herzog, 1992; Herzog and Gale, 1996; Herzog and Leverich, 2003; Herzog and Bryce, 2007). We constructed four items for measuring preference as there was not a specific scale for it. Four IRIs were also included for attention check. The IRI was: “*For this statement, please select [Much less/Less/Slightly less/About the same/Slightly more/More/Much more].*” All four IRIs had to be answered correctly for a participant’s responses to be considered as valid and included in the data analysis. The overall order of items was randomized for every participant.

**TABLE 4:** Scale items.

*Visitability*
I will stop at this place if I happen to be passing by.
I will walk out of my way to visit and spend time in this place.
I would regularly visit this place.
This is a place where I would choose to meet a friend.
*Restorativeness*
In this place, I would be able to concentrate well.
In this place, I would be able to focus on myself.
In this place, I would be able to relax.
In this place, I would be able to release all tension.
In this place, my energy level would get renewed.
*Preference*
I like this place a great deal.
I like this place very much.
I would enjoy this place a lot.
I would really enjoy this place.

### Study 1 Results

#### Composite Scores of Changes in Visitability, Restorativeness, and Preference

Using simple unit weighting, composite scores were computed to represent the changes in visitability, restorativeness, and preference of public space. The Cronbach’s alphas were 0.88 for change in visitability, 0.89 for change in restorativeness, and 0.93 for change in preference. There were significant positive correlations between changes in visitability and restorativeness (*r* = 0.81, *p* < 0.001), changes in visitability and preference (*r* = 0.89, *p* < 0.001), and changes in restorativeness and preference (*r* = 0.82, *p* < 0.001). Table 5 presents the mean changes in each variable across the 12 types of public space.

**TABLE 5:** Data from Study 1: Mean changes in visitability, restorativeness, and preference of public space as a function of street performance.

**Space type**	**Change in visitability**		**One-sample *t* test**		
	***M***	***SD***	***t***	***df***	***p***
All space types	0.63*	1.27	13.72	747	0.000
Transport facility	0.51*	1.27	3.30	66	0.002
Street	0.88*	0.99	7.36	68	0.000
Square	1.02*	0.98	8.29	62	0.000
Recreational space	0.38*	1.29	2.42	65	0.018
Found neighborhood space	0.41*	1.16	2.63	55	0.011
Park	0.61*	1.32	3.64	61	0.001
Memorial	–0.36	1.62	–1.74	60	0.087
Market	0.79*	1.20	4.89	55	0.000
Playground	0.70*	1.22	4.72	66	0.000
Community open space	0.97*	1.16	7.04	71	0.000
Indoor marketplace	0.56*	1.04	3.91	52	0.000
Waterfront	1.09*	1.24	6.61	55	0.000

**Space type**	**Change in restorativeness**		**One-sample *t* test**		
	***M***	***SD***	***t***	***df***	***p***

All space types	0.38*	1.27	8.08	747	0.000
Transport facility	0.28	1.28	1.80	66	0.077
Street	0.56*	1.05	4.45	68	0.000
Square	0.66*	1.10	4.76	62	0.000
Recreational space	0.33	1.34	1.98	65	0.052
Found neighborhood space	0.28	1.13	1.84	55	0.071
Park	0.34*	1.25	2.15	61	0.036
Memorial	–0.32	1.55	–1.58	60	0.119
Market	0.32	1.27	1.88	55	0.066
Playground	0.45*	1.27	2.92	66	0.005
Community open space	0.57*	1.31	3.70	71	0.000
Indoor marketplace	0.29	1.13	1.88	52	0.066
Waterfront	0.67*	1.27	3.94	55	0.000

**Space type**	**Change in preference**		**One-sample *t* test**		
	***M***	***SD***	***t***	***df***	***p***

All space types	0.78*	1.32	16.09	747	0.000
Transport facility	0.78*	1.27	5.02	66	0.000
Street	1.06*	0.97	9.11	68	0.000
Square	1.09*	1.05	8.26	62	0.000
Recreational space	0.52*	1.37	3.10	65	0.003
Found neighborhood space	0.48*	1.18	3.03	55	0.004
Park	0.73*	1.36	4.25	61	0.000
Memorial	–0.30	1.75	–1.35	60	0.181
Market	0.91*	1.20	5.64	55	0.000
Playground	0.77*	1.28	4.90	66	0.000
Community open space	1.16*	1.27	7.75	71	0.000
Indoor marketplace	0.90*	1.20	5.45	52	0.000
Waterfront	1.15*	1.16	7.41	55	0.000

*, statistically significant; *M*, mean change on a 7-point scale; *SD*, standard deviation; *t*, *t* statistic; *df*, degrees of freedom; and *p*, *p* value.

#### Hypothesis Testing

A series of one-sample *t* tests was performed to test the null hypotheses that the presence of street performance did not change the visitability (H1), restorativeness (H2), and preference (H3) of public space. First, we tested the hypotheses with the data of all 12 space types combined. We found that street performance significantly enhanced the perception of public space. Participants reported that street performance made the public spaces appear more visitable [*M* = 0.63, *SD* = 1.27, *t*(747) = 13.72, and *p* < 0.001], more restorative [*M* = 0.38, *SD* = 1.27, *t*(747) = 8.08, and *p* < 0.001], and more preferable [*M* = 0.78, *SD* = 1.32, *t*(747) = 16.09, and *p* < 0.001]^1^. Thus, H1, H2, and H3 were all supported.

We then conducted the same analysis with the data of the 12 space types split. Complete results are reported in Table 5. Generally speaking, we found that street performance significantly enhanced visitability and preference in all space types except memorial, and that it significantly enhanced restorativeness in half the space types – street, square, park, playground, community open space, and waterfront.

**FIGURE 3:**
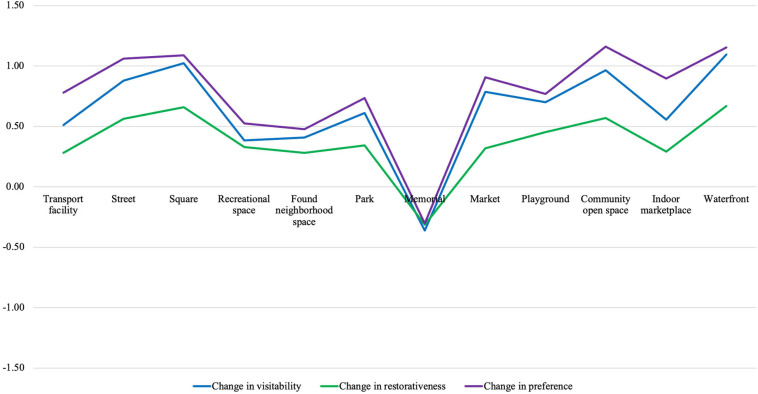
Effects of street performance across the 12 public spaces.

We noticed that street performance could also lead to negative changes in visitability, restorativeness, and preference, but that only applied to memorial. As presented in Figure 3, street performance only undermined the perception of memorial while it enhanced the perception of all the other 11 space types. But despite this opposite pattern, none of the negative effects of street performance on the perception of memorial reached statistical significance.

#### Effects of Street Performance Across the 12 Public Spaces

Although it did not reach statistical significance, the tendency that street performance undermined the perception of memorial led us to explore if the effects of street performance varied across the 12 public space types. A multivariate analysis of variance was conducted to test the null hypothesis that the changes in visitability, restorativeness, and preference were not different across the 12 space types. A significant overall difference was found [*F*(33, 2,163) = 3.20, *p* < 0.001, Wilk’s Λ = 0.87, and partial η^2^ = 0.05]. Univariate analyses of variance found that street performance changed the perceptions of the 12 space types differently, in terms of visitability [*F*(11, 736) = 6.37, *p* < 0.001, and partial η^2^ = 0.09], restorativeness [*F*(11, 736) = 2.68, *p* = 0.002, and partial η^2^ = 0.04], and preference [*F*(11, 736) = 6.35, *p* < 0.001, and partial η^2^ = 0.09]. We conducted a series of Tukey’s HSD *post hoc* tests to observe how the 12 space types differed among themselves in terms of the effects of street performance. Complete results are provided in Supplementary Appendix B. Since we are primarily interested in how the effects of street performance in memorial differed from those in the other space types, we present in Table 6 the comparison of memorial against the other space types in terms of the effects of street performance. Generally speaking, we found that the changes in the perception of memorial were consistently significantly lower than the changes in the perception of street, square, playground, community open space, and waterfront. This suggests that street performance could systematically enhance or undermine the perception of some space types but not others.

**TABLE 6:** Data from Study 1: Tukey’s HSD *post hoc* tests for comparing memorial against other space types in terms of the effects of street performance.

	**Mean difference**	**Standard error**	***p* value**
**Change in visitability**			
Transport facility	−0.87*	0.22	0.003
Street	−1.24*	0.21	0.000
Square	−1.38*	0.22	0.000
Recreational space	−0.74*	0.22	0.030
Found neighborhood space	−0.77*	0.23	0.034
Park	−0.97*	0.22	0.001
Market	−1.15*	0.23	0.000
Playground	−1.06*	0.22	0.000
Community open space	−1.33*	0.21	0.000
Indoor marketplace	−0.92*	0.23	0.004
Waterfront	−1.45*	0.23	0.000
**Change in restorativeness**			
Transport facility	–0.60	0.22	0.237
Street	−0.88*	0.22	0.004
Square	−0.98*	0.23	0.001
Recreational space	–0.64	0.22	0.149
Found neighborhood space	–0.59	0.23	0.306
Park	–0.66	0.23	0.141
Market	–0.63	0.23	0.215
Playground	−0.77*	0.22	0.028
Community open space	−0.88*	0.22	0.003
Indoor marketplace	–0.61	0.24	0.298
Waterfront	−0.98*	0.23	0.002
**Change in preference**			
Transport facility	−1.08*	0.22	0.000
Street	−1.36*	0.22	0.000
Square	−1.39*	0.23	0.000
Recreational space	−0.83*	0.23	0.014
Found neighborhood space	−0.78*	0.23	0.043
Park	−1.04*	0.23	0.000
Market	−1.21*	0.23	0.000
Playground	−1.07*	0.22	0.000
Community open space	−1.46*	0.22	0.000
Indoor marketplace	−1.20*	0.24	0.000
Waterfront	−1.46*	0.23	0.000

*Statistically significant.

### Study 1 Summary

Study 1 evaluated the extent to which the presence of street performance could change the perception of public space. We found that street performance made public space in general appear significantly more visitable (H1), more restorative (H2), and more preferable (H3). We also noticed that street performance tended to have a negative effect on the perception of memorial, although such an effect did not reach statistical significance. When comparing among the 12 space types, however, the effects of street performance on the perception of memorial were significantly lower than those of five other space types. This finding reveals that the effect of street performance might differ depending on public space type. Overall, Study 1 provides experimental support for the view that street performance can enhance people’s perception of public space.

Nonetheless, Study 1 has several limitations. First, we only used pictorial stimuli to simulate public spaces with street performance. The current study lacked mundane realism and complexity in simulating environmental experiences and the current findings might not readily generalize to the public spaces in reality. Second, the research participants took part in the study online and they had not physically visited the public spaces they were presented with. The participants were only responding to the study according to their imagination but not their actual experience of those spaces. Finally, the participants were asked explicitly about how the presence of street performance would change their perception of the public spaces. This could have been a strong experimental demand that had led them to guess the research purpose of the study and so respond more positively to the effect of street performance.

To address the abovementioned inadequacies, we conducted Study 2 – a between-group quasi-experiment in an actual public space – where the perceptions of the public space with vs. without street performance were surveyed from people physically present in the space.

## Study 2: Between-Group Quasi-Experiment of the Effect of Street Performance

We conducted a between-group quasi-experiment in an actual public space to examine the effect of street performance in a way that would address the various limitations of Study 1. Besides the initial hypotheses (H1, H2, and H3), we will also examine if the effects of street performance differ between two types of individuals: (a) engaged audience who have stopped to watch a street performance in a public space and (b) disengaged passersby who merely pass by a street performance in a public space. According to Leder et al. (2004), appreciation of an art object requires that the viewer of the object consciously identifies the object under question as art. In the context of street performance and in reality, in a public space where there is a street performance happening, people passing through the space may or may not consider the performance as art and they may or may not stop to engage with the performance. Thus, within the same public space where a street performance is present, engaged audience and disengaged passersby can be differentiated from each other. We theorize that engaged audience have a genuine connection with the street performance while they experience the public space whereas disengaged passersby have no or minimal connection with the street performance while they experience the public space. And we speculate that street performance affects the perception of public space differently between the two groups. In other words, the effect of street performance on the perception of public space may depend on whether or not a person is an engaged audience or a disengaged passerby. Hence, we further hypothesize that:

Relative to disengaged passersby, engaged audience will perceive a public space as more visitable (H4), more restorative (H5), and more preferable (H6).

### Study 2 Method

#### Study Location of Public Space With and Without Street Performance

**FIGURE 4:**
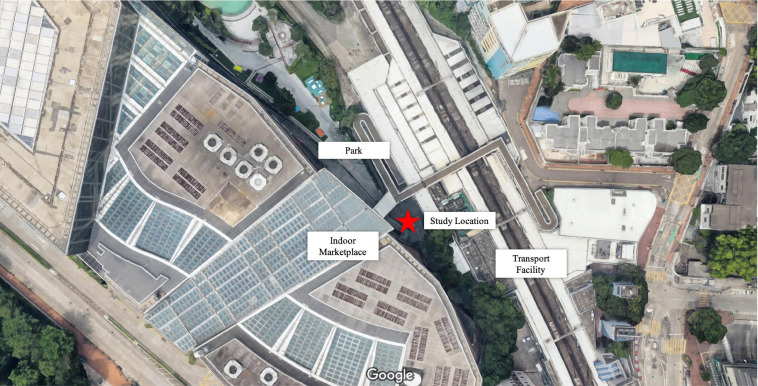
Map view of quasi-experiment location of Study 2.

**FIGURE 5:**
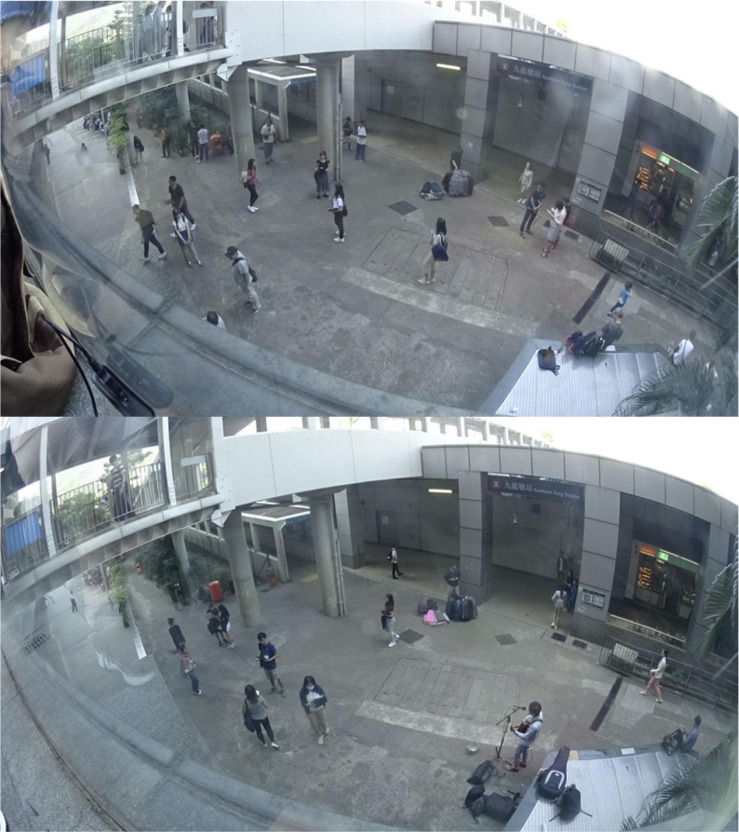
Quasi-experiment location without (top) and with (bottom) street performance.

We conducted a between-group quasi-experiment in a public space in Hong Kong where we manipulated the presence of street performance. We chose a public space in Kowloon Tong (see Figure 4; Google, n.d.) that would represent a mixture of four of the 12 major public space types (see Table 1). The chosen space was a found neighborhood space situated among a posh shopping mall (indoor marketplace), a small park, and a metro interchange station (transport facility). Thus, the space evidently did not only serve a single function. In the space we designated an area that measured approximately 30 m in length and 15 m in width. The area was typically frequented by local people and constant foot traffic between the shopping mall, the park, and the metro station. We carried out street survey in the area in two separate sessions: one without street performance (the control condition) and one with street performance (the experimental condition). Figure 5 shows the area in each session. The sessions took place on Sundays November 3 and 10, 2019, both between 14:00 and 17:30. It was typical early-fall air temperature with zero rainfalls on both dates^2^.

In the control condition on November 3, there was no street performance. In the experimental condition on November 10, we set up a musical-busking performance. The first author served the role of the busker; he had a Master of Art degree, training in the performing arts, and over 4 years of busking experience in Hong Kong. During the session, the busker sang and played an acoustic guitar at the same time. The singing was accompanied by strumming on the guitar. Both the voice and the guitar were amplified through a portable amplifier at a constant volume level. The busker sang Cantopop songs; Cantopop (short for Cantonese popular music) is the genre of popular music that features Western pop melodies and lyrics written in standard modern Chinese but sung in Cantonese (Chu and Leung, 2013, p. 65). Cantopop songs are typically in verse-chorus form and last 4–5 min each. Hong Kong popular music is strongly identified with Cantopop (Chu, 2017). Thus, the music of the street performance of the current study should represent a familiar genre among the Hong Kong locals. An open guitar case was placed at the busker’s foot intended as a receptacle for donations from passersby.

#### Participants

People passing through the designated area were intercepted and invited to take part in the study. Six research assistants constantly observed the area throughout the sessions. People who had entered the area were identified as potential research participants; as they left the area they were approached and invited by one of the research assistants.

We approached 470 and 419 people in the control and experimental sessions, respectively. A total of 162 responses were collected: 88 (54.3%) in the control session and 74 (45.7%) in the experimental session; thus, survey response rates were 18.7% and 17.7%, respectively. Each participant received HK$20 (approximately US$2.55) for participation. Within the experimental session, there were 30 engaged audience (40.5%) and 44 disengaged passersby (59.5%).

The entire sample comprised 66 women and 94 men (2 preferred not to answer). Majority of the sample aged between 18 and 24 years (43.8%); about a fifth aged between 25 and 29 years (19.1%) and between 30 and 39 years (20.4%); smaller proportions aged between 40 and 49 years (6.8%), between 50 and 59 years (5.6%), and 60 years or above (1.9%); and four participants preferred not to report their age. Table 7 presents the sample’s demographics. Majority of the sample (78.4%) reported that they had lived in Hong Kong for at least 7 years; nine participants for less than 1 year (5.6%) and five were travelers (3.1%). Proportions were about the same between those who had attained a bachelor’s degree as their highest education level (38.3%) and those who had not (43.8%), and the rest had attained an education level above a bachelor’s degree (15.4%). Most participants were only passing through the study location (43.2%); a fair proportion had stayed in the location for up to 15 min (36.4%), fewer up to 1 h (11.1%), and the rest more than 1 h (9.2%). Majority reported that they had been to the study location for more than 40 times (32.1%); there were also first-timers (4.9%); and those who had only been there for one to two times (11.1%); the rest were in between (51.8%).

**TABLE 7:** Demographics of Study 2 sample.

	***n***	**%**
**Length of residence in Hong Kong**		
Traveler	5	3.1
Less than 1 year	9	5.6
At least 1 year	8	4.9
At least 2 years	2	1.2
At least 3 years	3	1.9
At least 4 years	2	1.2
At least 7 years	127	78.4
Prefer not to answer	6	3.7
**Education**		
Primary or below	1	0.6
Secondary	41	25.3
Post-secondary: diploma/certificate	24	14.8
Associate’s degree	5	3.1
Bachelor’s degree	62	38.3
Master’s degree	23	14.2
Doctoral degree	2	1.2
Prefer not to answer	4	2.5
**Current time spent in study location**		
No stopover at all (i.e., walking through)	70	43.2
Up to 15 min	59	36.4
Up to 1 h	18	11.1
Up to 2 h	7	4.3
Up to 3 h	1	0.6
Up to 4 h	2	1.2
More than 4 h	5	3.1
**Prior visit to study location**		
First time	8	4.9
1–2 times	18	11.1
Multiple times	31	19.1
More than 10 times	28	17.3
More than 20 times	12	7.4
More than 30 times	13	8.0
More than 40 times	52	32.1

#### Procedure

After giving their informed consent, research participants were given a paper questionnaire to fill out on the spot. The survey began with an introduction that explicitly stated that the study was to understand human experience of public space. Participants were asked to evaluate their perception of the designated area (i.e., the public space at the intersection between the shopping mall, the park, and the metro station). Participants were reminded to focus on the specified area only and that there were no right or wrong answers.

#### Measures

Participants reported their perception of the public space in terms of visitability, restorativeness, and preference on a 7-point Likert scale (from *strongly disagree* to *strongly agree* coded from 1 to 7 with the midpoint *neither agree nor disagree* as 4). Scale items were the same as Study 1 (see Table 4) except that they were back-translated into Chinese by two postgraduate psychology students. Chinese was an official language of Hong Kong; over 90% of the Hong Kong population spoke Chinese (GovHK, 2020). Thus, when conducting street survey in Hong Kong, a respondent should most likely be most familiar with the Chinese language. In the study, both the initial English version and the back-translated Chinese version were available. Eventually, 90.7% of our sample chose the Chinese version, and 9.3% chose the English version. All back-translated items are provided in Supplementary Appendix C. Questionnaire items were presented in blocks, each block corresponded to one variable. The order of the variables was: restorativeness, visitability, and preference. The order of the items was the same for all participants.

### Study 2 Results

#### Composite Scores of Visitability, Restorativeness, and Preference

Using simple unit weighting, composite scores were computed to represent visitability, restorativeness, and preference. The Cronbach’s alphas were 0.81 for visitability, 0.91 for restorativeness, and 0.96 for preference. There were significant positive correlations between visitability and restorativeness (*r* = 0.47, *p* < 0.001), visitability and preference (*r* = 0.64, *p* < 0.001), and restorativeness and preference (*r* = 0.57, *p* < 0.001). Table 8 presents the mean ratings of each variable in each group.

**TABLE 8:** Data from Study 2: Mean ratings of visitability, restorativeness, and preference of public space.

**Visitability**					
**Control group**		**Experimental group**			
***M***	***SD***	***M***		***SD***	

3.78	1.29	3.68		1.30	

		**Disengaged passersby**		**Engaged audience**	
		***M***	***SD***	***M***	***SD***

		3.31	1.34	4.23	1.05

**Restorativeness**					

**Control group**		**Experimental group**			
***M***	***SD***	***M***		***SD***	

3.80	1.08	4.19		1.22	

		**Disengaged passersby**		**Engaged audience**	
		***M***	***SD***	***M***	***SD***

		3.96	1.17	4.51	1.24

**Preference**					

**Control group**		**Experimental group**			
***M***	***SD***	***M***		***SD***	

4.13	1.19	4.27		1.17	

		**Disengaged passersby**		**Engaged audience**	
		***M***	***SD***	***M***	***SD***

		4.02	1.23	4.64	0.97

**M*, group mean and *SD*, standard deviation.*

#### Hypothesis Testing

A set of two orthogonal contrasts compared the perceptions of the public space (a) between the experimental group (engaged audience and disengaged passersby combined) and the control group (C1: engaged audience = 1, disengaged passersby = 1, and control group = −2) and (b) between the engaged audience and the disengaged passersby (C2: engaged audience = 1, disengaged passersby = −1, and control group = 0).

Compared to the control group, the experimental group perceived the public space as significantly more restorative [*M*s = 4.19 vs. 3.80, *t*(159) = 2.44, and *p* = 0.016], but their perception did not differ significantly in visitability [*M*s = 3.68 vs. 3.78, *t*(159) = −0.06, and *p* = 0.951] nor preference [*M*s = 4.27 vs. 4.13, *t*(159) = 1.09, *p* = 0.280]. Thus, H2 was supported but H1 and H3 were not.

Compared to the disengaged passersby, the engaged audience perceived the public space as significantly more visitable [*M*s = 4.23 vs. 3.31, *t*(159) = 3.05, *p* = 0.003], more restorative [*M*s = 4.51 vs. 3.96, *t*(159) = 2.05, *p* = 0.042], and more preferable [*M*s = 4.64 vs. 4.02, *t*(159) = 2.24, *p* = 0.026]. Thus, H4, H5, and H6 were all supported.

### Study 2 Summary

Study 2 was a between-group quasi-experiment that examined the effect of street performance on the perception of an actual public space in Hong Kong. We found that street performance could make the public space significantly more restorative, supporting H2. We did not find significant results regarding visitability (H1) and preference (H3). However, when comparing between engaged audience’s and disengaged passersby’s perceptions of the public space with a street performance happening, we found that the engaged audience perceived the space as significantly more visitable (H4), more restorative (H5), and more preferable (H6) than did the disengaged passersby. This finding suggests that the effect of street performance may be more prominent among people who have engaged with street performance. Overall, Study 2 provides further experimental support for the view that street performance can enhance people’s perception of public space.

## General Discussion

### Summary and Major Findings

To the best of our knowledge, this is the first study to take an experimental approach to test the effect of street performance on the perception of public space. In the literature where street performance is concerned, it has long been established that street performance can enhance people’s experience of public space, but the studies advocating such a view were limited to either observations or descriptive surveys. To address the current research gap, we conducted two experimental studies to verify the effect of street performance. Study 1 was an online computer-based study where research participants evaluated the extent to which the presence of street performance would change their perception of 12 major types of public space. We found that street performance improved the visitability, restorativeness, and preference of public spaces in general. Study 2 was a between-group quasi-experiment in an actual public space where we manipulated the presence of street performance and surveyed people passing through the space about their perception of the space. We found that public space was perceived as more restorative with street performance than without street performance. In addition, people who had engaged with street performance in the space perceived the space as more visitable, more restorative, and more preferable than people who had not engaged with street performance. In summary, both Studies 1 and 2 provide experimental support for the view that street performance can enhance people’s experience of public space. In the broader research context, our findings confirm the effect of music on our behavior in everyday situation – music can impact how we perceive and interact with our immediate environment.

Studies 1 and 2 took different but complementary approaches in examining the effect of street performance on the perception of public space. They differed in terms of the sensory modality through which the research participants experienced and evaluated the various settings of public space. Study 1 participants were only presented with visual images and so they only engaged with public space through a single modality. Study 2 participants were physically present in an actual public space and so they engaged with public space through multiple modalities. In terms of the effect of musical busking, Study 1 participants did not hear the sound of the performance whereas Study 2 participants could actually hear the sound of the performance. Hence, Study 1 participants responded based on their own imagination and expectations about street performance whereas Study 2 participants responded based on their actual experience with the street performance that was (not) taking place in the public space. In other words, findings of Study 1 could reflect more people’s general beliefs and expectations about how street performance would impact their perception of public space whereas findings of Study 2 could reflect more the result driven by the presence vs. absence of street performance in a public space. Studies 1 and 2 could have been treating separate aspects of the effect of street performance on public space, but they are demonstrably *not* contradictory. We believe that the two studies provide complementary evidences regarding the effect of street performance on the perception of public space.

In this paper we operationalize public space on the basis of a theoretical typology built upon previous reviews of public space (Carr et al., 1992; Gehl and Gemzøe, 2001; Stanley et al., 2012; Ho and Au, 2020). Following such a typology, Study 1 examined the effect of street performance with respect to 12 major public space types and Study 2 was conducted in a location that approximated a mixture of four major public space types. Thus, we did not limit our investigation to a single space type but instead we had considered multiple space types. While we are mindful about the limitation in how we represented the vast notion of public space, we believe that the current findings should be relevant to understanding the impact of street performance on the most common types of public spaces.

Concerning Study 2, an alternative explanation can be made regarding the effect of street performance between engaged audience and disengaged passersby. Such a finding could have merely reflected certain individual qualities instead of the effect of street performance. It is possible that individuals who were more interested in the arts and entertainment were more likely to stop to watch a street performance and were also more likely to perceive public space in a positive light, as compared with individuals who were less interested in the arts and entertainment. In other words, the differences in the perception of public space between engaged audience and disengaged passersby could have been attributed to their individual preferences toward the arts and entertainment rather than their (dis)engagement with street performance. Still, the current interpretation is totally valid. Future studies may control for individual factors and dispositions to clarify the effect of street performance between engaged audience and disengaged passersby.

The current findings have some practical implications. As we have pointed out at the beginning, the legitimacy of street performance is often challenged in reality. Street performance is not universally acceptable; it is legal in some places but illegal in others. Legality of street performance essentially depends on whether street performance is seen as desirable or undesirable in public space. The role of the policy makers is to determine if street performance can improve or undermine the quality of public space. The present study sheds light on this practical issue. Our studies found that street performance could enhance the perception of public spaces of the most common types. Therefore, we would recommend that street performance is a beneficial feature to public space, and we encourage policy makers to promote street performance to the general public.

### Limitations

The current findings are limited by a narrow representation of street performance. Despite the diversity of street performance, we restricted our operationalization to musical busking only. And at the stimulus level, we adopted only a single form of musical busking. Thus, any effects of street performance observed in the current study could be attributable only to our selected operationalization and representation of street performance. The current findings might not readily generalize to some other situations where different performance types are concerned. Street performance can be divided into musical and non-musical, which differ in how they typically occupy public space. Musical busking is typically characterized by its sonic properties; a fixed and close observation of the performance is often unnecessary in appreciating musical busking. Non-musical busking is typically characterized by its visual appeals; a fixed and close observation of the performance is often necessary in appreciating non-musical busking. In other words, musical busking usually allows its spectators to enjoy the performance from a distance or as they pass through a public space whereas non-musical busking usually demands its spectators to stop and gather around the performance if they wish to enjoy the performance. As musical and non-musical busking occupy public space differently, it is possible that they also impact people’s perception of public space differently. Future studies should take this into account in validating the effect of street performance.

Regarding Study 2, the representativeness of our sample could have been limited by the selected study location. While the research participants were fairly evenly distributed in terms of gender, age group, and education, a large proportion (75.93%) reported that they had lived in Hong Kong for at least 7 years and that they had prior experience with the location (i.e., they were not first-timers). In other words, majority of the sample was local residents to whom the study location and cultural context were not novel; the current findings would best represent the experience of individuals evaluating a public space they were already familiar with. Effect of street performance could have been different had our sample comprised mostly non-local visitors or travelers instead. Future studies may consider examining the effect of street performance on visitors’ or travelers’ perception of specific touristic locations.

Finally, our investigation focused only on a hypothetical, socioculturally neutral street performance. We had not considered the sociocultural factors that might influence the perception of street performance. Depending on the cultural context, the desirability of street performance may succumb to social prejudices. Street performers may be associated with some stigmatized groups, and certain forms of street performance may be seen as threatening or intrusive to some people. More often than not, street performers are associated with beggars. Since street performers typically do not hold stable, full-time jobs like normal people do, they may be labeled as unemployed or unproductive individuals who fail to meet societal expectations. At other times, certain forms of street performance may be seen as threatening for involving hazardous acts (e.g., fire-eating, stunt shows, and juggling of sharp objects, etc.) or intrusive for containing explicit political contents (Mason, 1992; Cohen-Cruz, 1998; Martin, 2004). Sociocultural factors as such play a role in determining the desirability of street performance in the public space. While the selection of a socioculturally neutral street performance as in the present paper allows us to postulate a standard effect of street performance on the perception of public space, it cannot account for the sociocultural influence. Future studies should consult a cross-cultural perspective to clarify the effect of street performance on public space.

## Data Availability Statement

The raw data supporting the conclusions of this article will be made available by the authors, without undue reservation.

## Ethics Statement

The studies involving human participants were reviewed and approved by The Survey and Behavioral Research Ethics Committee of The Chinese University of Hong Kong. The patients/participants provided their written informed consent to participate in this study.

## Author Contributions

RH was the principal investigator of this research and was responsible for creating the main content of this publication. WTA was a co-investigator and was involved in the design, methodology, analysis, and reporting of this publication. Both authors contributed to the article and approved the submitted version.

## Conflict of Interest

The authors declare that the research was conducted in the absence of any commercial or financial relationships that could be construed as a potential conflict of interest.

## Footnotes

1 *M* expresses mean change.

2 Over the 10 years prior to the current study, the historical air temperature of the period between November 3 and 10 in Hong Kong ranged from 22 to 26°C and averaged 24°C (*SD* = 1.4°C; Hong Kong Observatory, n.d.). The air temperatures in the two study sessions were 26 and 23°C, respectively; thus, the average air temperature across the entire study period was 24°C, which was 0.3 *SD* higher than the historical average and within the historical range.
