# Supplementary material for: Effect of Street Performance (Busking) on the Environmental Perception of Public Space
Source: Front Psychol. 2021 Mar 30;12:647863. doi: 10.3389/fpsyg.2021.647863 (PMC8042224; doi:10.3389/fpsyg.2021.647863)
Supplement: Supplementary file 1 [file Presentation_1.PPTX]

## Slide 1
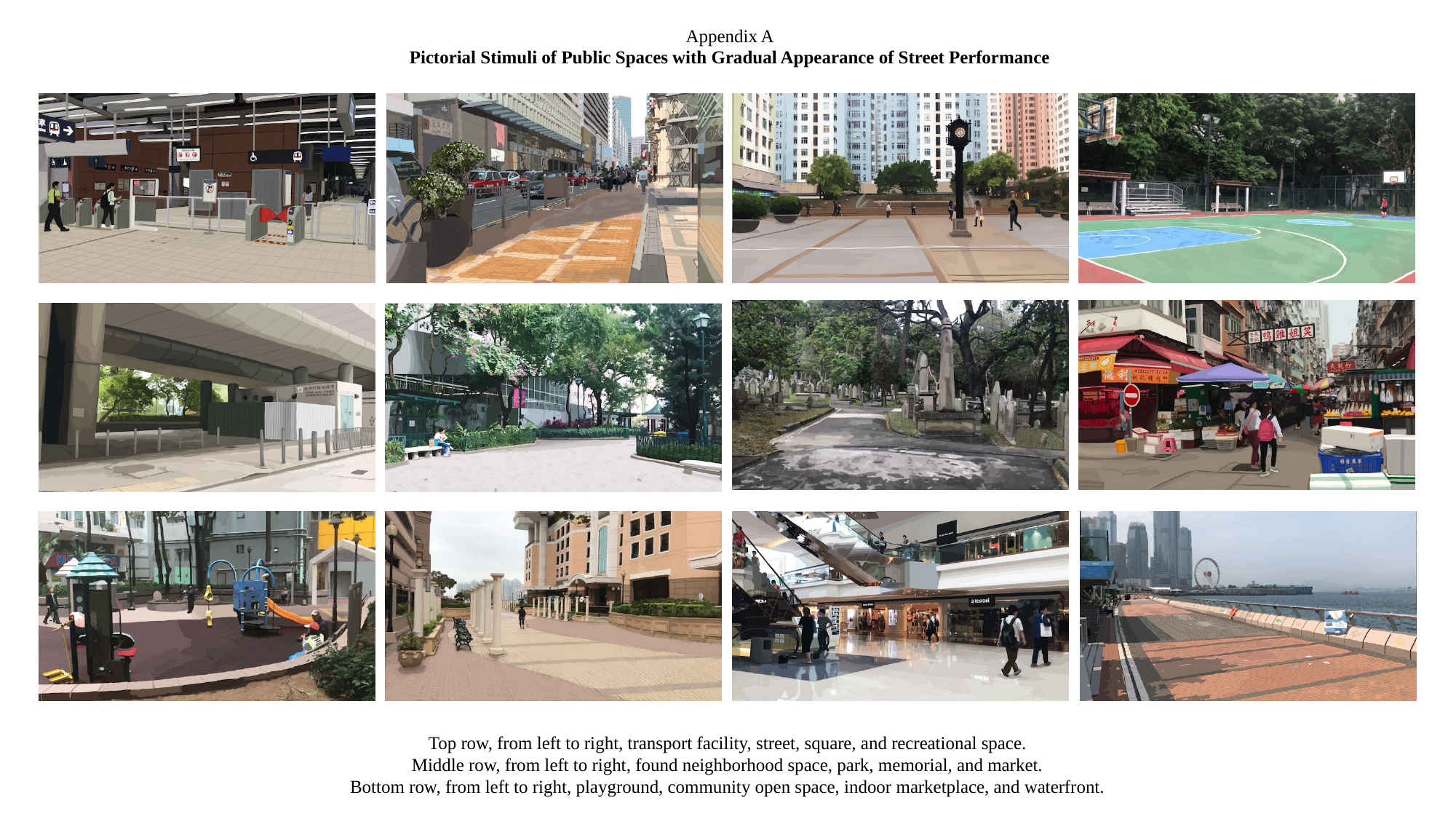

Appendix A
Pictorial Stimuli of Public Spaces with Gradual Appearance of Street Performance
Top row, from left to right, transport facility, street, square, and recreational space.
Middle row, from left to right, found neighborhood space, park, memorial, and market.
Bottom row, from left to right, playground, community open space, indoor marketplace, and waterfront.
