# Supplementary material for: Effect of Street Performance (Busking) on the Environmental Perception of Public Space
Source: Front Psychol. 2021 Mar 30;12:647863. doi: 10.3389/fpsyg.2021.647863 (PMC8042224; doi:10.3389/fpsyg.2021.647863)
Supplement: Supplementary file 2 [file Data_Sheet_2.pdf]

Appendix B

Multiple Comparisons with Tukey's HSD Post-hoc Tests

Change in Visitability (1 of 3)

Space type	Comparison space type	Mean difference	Standard error	*p* value
Transport facility	Street	-0.37	0.21	0.835
	Square	-0.51	0.21	0.408
	Recreational space	0.13	0.21	1.000
	Found neighborhood space	0.10	0.22	1.000
	Park	-0.10	0.21	1.000
	Memorial	0.87	0.22	0.003
	Market	-0.27	0.22	0.985
	Playground	-0.19	0.21	0.999
	Community open space	-0.45	0.21	0.552
	Indoor marketplace	-0.05	0.22	1.000
	Waterfront	-0.58	0.22	0.258
Street	Transport facility	0.37	0.21	0.835
	Square	-0.14	0.21	1.000
	Recreational space	0.50	0.21	0.425
	Found neighborhood space	0.47	0.22	0.576
	Park	0.27	0.21	0.982
	Memorial	1.24	0.21	0.000
	Market	0.09	0.22	1.000
	Playground	0.18	0.21	0.999
	Community open space	-0.08	0.21	1.000
	Indoor marketplace	0.32	0.22	0.952
	Waterfront	-0.21	0.22	0.998
Square	Transport facility	0.51	0.21	0.408
	Street	0.14	0.21	1.000
	Recreational space	0.64	0.21	0.114
	Found neighborhood space	0.62	0.22	0.199
	Park	0.41	0.22	0.756
	Memorial	1.38	0.22	0.000
	Market	0.24	0.22	0.996
	Playground	0.32	0.21	0.938
	Community open space	0.06	0.21	1.000
	Indoor marketplace	0.47	0.23	0.653
	Waterfront	-0.07	0.22	1.000
Recreational space	Transport facility	-0.13	0.21	1.000
	Street	-0.50	0.21	0.425
	Square	-0.64	0.21	0.114
	Found neighborhood space	-0.02	0.22	1.000
	Park	-0.23	0.22	0.996
	Memorial	0.74	0.22	0.030
	Market	-0.40	0.22	0.805
	Playground	-0.32	0.21	0.938
	Community open space	-0.58	0.21	0.179
	Indoor marketplace	-0.17	0.22	1.000
	Waterfront	-0.71	0.22	0.061

Change in Visitability (2 of 3)

Space type	Comparison space type	Mean difference	Standard error	*p* value
Found neighborhood space	Transport facility	-0.10	0.22	1.000
	Street	-0.47	0.22	0.576
	Square	-0.62	0.22	0.199
	Recreational space	0.02	0.22	1.000
	Park	-0.20	0.22	0.999
	Memorial	0.77	0.23	0.034
	Market	-0.38	0.23	0.890
	Playground	-0.30	0.22	0.974
	Community open space	-0.56	0.22	0.295
	Indoor marketplace	-0.15	0.23	1.000
	Waterfront	-0.69	0.23	0.114
Park	Transport facility	0.10	0.21	1.000
	Street	-0.27	0.21	0.982
	Square	-0.41	0.22	0.756
	Recreational space	0.23	0.22	0.996
	Found neighborhood space	0.20	0.22	0.999
	Memorial	0.97	0.22	0.001
	Market	-0.18	0.22	1.000
	Playground	-0.09	0.21	1.000
	Community open space	-0.36	0.21	0.873
	Indoor marketplace	0.05	0.23	1.000
	Waterfront	-0.48	0.22	0.579
Memorial	Transport facility	-0.87	0.22	0.003
	Street	-1.24	0.21	0.000
	Square	-1.38	0.22	0.000
	Recreational space	-0.74	0.22	0.030
	Found neighborhood space	-0.77	0.23	0.034
	Park	-0.97	0.22	0.001
	Market	-1.15	0.23	0.000
	Playground	-1.06	0.22	0.000
	Community open space	-1.33	0.21	0.000
	Indoor marketplace	-0.92	0.23	0.004
	Waterfront	-1.45	0.23	0.000
Market	Transport facility	0.27	0.22	0.985
	Street	-0.09	0.22	1.000
	Square	-0.24	0.22	0.996
	Recreational space	0.40	0.22	0.805
	Found neighborhood space	0.38	0.23	0.890
	Park	0.18	0.22	1.000
	Memorial	1.15	0.23	0.000
	Playground	0.08	0.22	1.000
	Community open space	-0.18	0.22	1.000
	Indoor marketplace	0.23	0.23	0.998
	Waterfront	-0.31	0.23	0.974

Change in Visitability (3 of 3)

Space type	Comparison space type	Mean difference	Standard error	*p* value
Playground	Transport facility	0.19	0.21	0.999
	Street	-0.18	0.21	0.999
	Square	-0.32	0.21	0.938
	Recreational space	0.32	0.21	0.938
	Found neighborhood space	0.30	0.22	0.974
	Park	0.09	0.21	1.000
	Memorial	1.06	0.22	0.000
	Market	-0.08	0.22	1.000
	Community open space	-0.26	0.21	0.982
	Indoor marketplace	0.14	0.22	1.000
	Waterfront	-0.39	0.22	0.829
Community open space	Transport facility	0.45	0.21	0.552
	Street	0.08	0.21	1.000
	Square	-0.06	0.21	1.000
	Recreational space	0.58	0.21	0.179
	Found neighborhood space	0.56	0.22	0.295
	Park	0.36	0.21	0.873
	Memorial	1.33	0.21	0.000
	Market	0.18	0.22	1.000
	Playground	0.26	0.21	0.982
	Indoor marketplace	0.41	0.22	0.787
	Waterfront	-0.13	0.22	1.000
Indoor marketplace	Transport facility	0.05	0.22	1.000
	Street	-0.32	0.22	0.952
	Square	-0.47	0.23	0.653
	Recreational space	0.17	0.22	1.000
	Found neighborhood space	0.15	0.23	1.000
	Park	-0.05	0.23	1.000
	Memorial	0.92	0.23	0.004
	Market	-0.23	0.23	0.998
	Playground	-0.14	0.22	1.000
	Community open space	-0.41	0.22	0.787
	Waterfront	-0.54	0.23	0.476
Waterfront	Transport facility	0.58	0.22	0.258
	Street	0.21	0.22	0.998
	Square	0.07	0.22	1.000
	Recreational space	0.71	0.22	0.061
	Found neighborhood space	0.69	0.23	0.114
	Park	0.48	0.22	0.579
	Memorial	1.45	0.23	0.000
	Market	0.31	0.23	0.974
	Playground	0.39	0.22	0.829
	Community open space	0.13	0.22	1.000
	Indoor marketplace	0.54	0.23	0.476

Change in Restorativeness (1 of 3)

Space type	Comparison space type	Mean difference	Standard error	*p* value
Transport facility	Street	-0.28	0.22	0.978
	Square	-0.38	0.22	0.856
	Recreational space	-0.05	0.22	1.000
	Found neighborhood space	0.00	0.23	1.000
	Park	-0.06	0.22	1.000
	Memorial	0.60	0.22	0.237
	Market	-0.04	0.23	1.000
	Playground	-0.17	0.22	1.000
	Community open space	-0.29	0.21	0.971
	Indoor marketplace	-0.01	0.23	1.000
	Waterfront	-0.39	0.23	0.865
Street	Transport facility	0.28	0.22	0.978
	Square	-0.10	0.22	1.000
	Recreational space	0.24	0.22	0.995
	Found neighborhood space	0.28	0.23	0.984
	Park	0.22	0.22	0.998
	Memorial	0.88	0.22	0.004
	Market	0.24	0.23	0.995
	Playground	0.11	0.22	1.000
	Community open space	-0.01	0.21	1.000
	Indoor marketplace	0.27	0.23	0.990
	Waterfront	-0.11	0.23	1.000
Square	Transport facility	0.38	0.22	0.856
	Street	0.10	0.22	1.000
	Recreational space	0.33	0.22	0.938
	Found neighborhood space	0.38	0.23	0.886
	Park	0.32	0.22	0.960
	Memorial	0.98	0.23	0.001
	Market	0.34	0.23	0.944
	Playground	0.21	0.22	0.999
	Community open space	0.09	0.22	1.000
	Indoor marketplace	0.37	0.23	0.915
	Waterfront	-0.01	0.23	1.000
Recreational space	Transport facility	0.05	0.22	1.000
	Street	-0.24	0.22	0.995
	Square	-0.33	0.22	0.938
	Found neighborhood space	0.05	0.23	1.000
	Park	-0.01	0.22	1.000
	Memorial	0.64	0.22	0.149
	Market	0.01	0.23	1.000
	Playground	-0.13	0.22	1.000
	Community open space	-0.24	0.21	0.993
	Indoor marketplace	0.04	0.23	1.000
	Waterfront	-0.34	0.23	0.942

Change in Restorativeness (2 of 3)

Space type	Comparison space type	Mean difference	Standard error	*p* value
Found neighborhood space	Transport facility	0.00	0.23	1.000
	Street	-0.28	0.23	0.984
	Square	-0.38	0.23	0.886
	Recreational space	-0.05	0.23	1.000
	Park	-0.06	0.23	1.000
	Memorial	0.59	0.23	0.306
	Market	-0.04	0.24	1.000
	Playground	-0.18	0.23	1.000
	Community open space	-0.29	0.22	0.979
	Indoor marketplace	-0.01	0.24	1.000
	Waterfront	-0.39	0.24	0.893
Park	Transport facility	0.06	0.22	1.000
	Street	-0.22	0.22	0.998
	Square	-0.32	0.22	0.960
	Recreational space	0.01	0.22	1.000
	Found neighborhood space	0.06	0.23	1.000
	Memorial	0.66	0.23	0.141
	Market	0.02	0.23	1.000
	Playground	-0.11	0.22	1.000
	Community open space	-0.23	0.22	0.997
	Indoor marketplace	0.05	0.23	1.000
	Waterfront	-0.33	0.23	0.961
Memorial	Transport facility	-0.60	0.22	0.237
	Street	-0.88	0.22	0.004
	Square	-0.98	0.23	0.001
	Recreational space	-0.64	0.22	0.149
	Found neighborhood space	-0.59	0.23	0.306
	Park	-0.66	0.23	0.141
	Market	-0.63	0.23	0.215
	Playground	-0.77	0.22	0.028
	Community open space	-0.88	0.22	0.003
	Indoor marketplace	-0.61	0.24	0.298
	Waterfront	-0.98	0.23	0.002
Market	Transport facility	0.04	0.23	1.000
	Street	-0.24	0.23	0.995
	Square	-0.34	0.23	0.944
	Recreational space	-0.01	0.23	1.000
	Found neighborhood space	0.04	0.24	1.000
	Park	-0.02	0.23	1.000
	Memorial	0.63	0.23	0.215
	Playground	-0.14	0.23	1.000
	Community open space	-0.25	0.22	0.993
	Indoor marketplace	0.03	0.24	1.000
	Waterfront	-0.35	0.24	0.946

Change in Restorativeness (3 of 3)

Space type	Comparison space type	Mean difference	Standard error	*p* value
Playground	Transport facility	0.17	0.22	1.000
	Street	-0.11	0.22	1.000
	Square	-0.21	0.22	0.999
	Recreational space	0.13	0.22	1.000
	Found neighborhood space	0.18	0.23	1.000
	Park	0.11	0.22	1.000
	Memorial	0.77	0.22	0.028
	Market	0.14	0.23	1.000
	Community open space	-0.12	0.21	1.000
	Indoor marketplace	0.16	0.23	1.000
	Waterfront	-0.21	0.23	0.999
Community open space	Transport facility	0.29	0.21	0.971
	Street	0.01	0.21	1.000
	Square	-0.09	0.22	1.000
	Recreational space	0.24	0.21	0.993
	Found neighborhood space	0.29	0.22	0.979
	Park	0.23	0.22	0.997
	Memorial	0.88	0.22	0.003
	Market	0.25	0.22	0.993
	Playground	0.12	0.21	1.000
	Indoor marketplace	0.28	0.23	0.987
	Waterfront	-0.10	0.22	1.000
Indoor marketplace	Transport facility	0.01	0.23	1.000
	Street	-0.27	0.23	0.990
	Square	-0.37	0.23	0.915
	Recreational space	-0.04	0.23	1.000
	Found neighborhood space	0.01	0.24	1.000
	Park	-0.05	0.23	1.000
	Memorial	0.61	0.24	0.298
	Market	-0.03	0.24	1.000
	Playground	-0.16	0.23	1.000
	Community open space	-0.28	0.23	0.987
	Waterfront	-0.38	0.24	0.919
Waterfront	Transport facility	0.39	0.23	0.865
	Street	0.11	0.23	1.000
	Square	0.01	0.23	1.000
	Recreational space	0.34	0.23	0.942
	Found neighborhood space	0.39	0.24	0.893
	Park	0.33	0.23	0.961
	Memorial	0.98	0.23	0.002
	Market	0.35	0.24	0.946
	Playground	0.21	0.23	0.999
	Community open space	0.10	0.22	1.000
	Indoor marketplace	0.38	0.24	0.919

Change in Preference (1 of 3)

Space type	Comparison space type	Mean difference	Standard error	*p* value
Transport facility	Street	-0.28	0.22	0.980
	Square	-0.31	0.22	0.967
	Recreational space	0.26	0.22	0.991
	Found neighborhood space	0.30	0.23	0.977
	Park	0.05	0.22	1.000
	Memorial	1.08	0.22	0.000
	Market	-0.13	0.23	1.000
	Playground	0.01	0.22	1.000
	Community open space	-0.38	0.22	0.837
	Indoor marketplace	-0.12	0.23	1.000
	Waterfront	-0.37	0.23	0.902
Street	Transport facility	0.28	0.22	0.980
	Square	-0.03	0.22	1.000
	Recreational space	0.54	0.22	0.363
	Found neighborhood space	0.58	0.23	0.305
	Park	0.33	0.22	0.947
	Memorial	1.36	0.22	0.000
	Market	0.16	0.23	1.000
	Playground	0.29	0.22	0.973
	Community open space	-0.10	0.21	1.000
	Indoor marketplace	0.17	0.23	1.000
	Waterfront	-0.09	0.23	1.000
Square	Transport facility	0.31	0.22	0.967
	Street	0.03	0.22	1.000
	Recreational space	0.56	0.22	0.325
	Found neighborhood space	0.61	0.23	0.272
	Park	0.35	0.23	0.924
	Memorial	1.39	0.23	0.000
	Market	0.18	0.23	1.000
	Playground	0.32	0.22	0.957
	Community open space	-0.07	0.22	1.000
	Indoor marketplace	0.19	0.24	1.000
	Waterfront	-0.06	0.23	1.000
Recreational space	Transport facility	-0.26	0.22	0.991
	Street	-0.54	0.22	0.363
	Square	-0.56	0.22	0.325
	Found neighborhood space	0.05	0.23	1.000
	Park	-0.21	0.22	0.999
	Memorial	0.83	0.23	0.014
	Market	-0.38	0.23	0.884
	Playground	-0.25	0.22	0.994
	Community open space	-0.64	0.22	0.127
	Indoor marketplace	-0.37	0.23	0.910
	Waterfront	-0.63	0.23	0.214

Change in Preference (2 of 3)

Space type	Comparison space type	Mean difference	Standard error	*p* value
Found neighborhood space	Transport facility	-0.30	0.23	0.977
	Street	-0.58	0.23	0.305
	Square	-0.61	0.23	0.272
	Recreational space	-0.05	0.23	1.000
	Park	-0.26	0.23	0.995
	Memorial	0.78	0.23	0.043
	Market	-0.43	0.24	0.825
	Playground	-0.29	0.23	0.983
	Community open space	-0.68	0.23	0.106
	Indoor marketplace	-0.42	0.24	0.858
	Waterfront	-0.67	0.24	0.177
Park	Transport facility	-0.05	0.22	1.000
	Street	-0.33	0.22	0.947
	Square	-0.35	0.23	0.924
	Recreational space	0.21	0.22	0.999
	Found neighborhood space	0.26	0.23	0.995
	Memorial	1.04	0.23	0.000
	Market	-0.17	0.23	1.000
	Playground	-0.03	0.22	1.000
	Community open space	-0.43	0.22	0.735
	Indoor marketplace	-0.16	0.24	1.000
	Waterfront	-0.42	0.23	0.825
Memorial	Transport facility	-1.08	0.22	0.000
	Street	-1.36	0.22	0.000
	Square	-1.39	0.23	0.000
	Recreational space	-0.83	0.23	0.014
	Found neighborhood space	-0.78	0.23	0.043
	Park	-1.04	0.23	0.000
	Market	-1.21	0.23	0.000
	Playground	-1.07	0.22	0.000
	Community open space	-1.46	0.22	0.000
	Indoor marketplace	-1.20	0.24	0.000
	Waterfront	-1.46	0.23	0.000
Market	Transport facility	0.13	0.23	1.000
	Street	-0.16	0.23	1.000
	Square	-0.18	0.23	1.000
	Recreational space	0.38	0.23	0.884
	Found neighborhood space	0.43	0.24	0.825
	Park	0.17	0.23	1.000
	Memorial	1.21	0.23	0.000
	Playground	0.14	0.23	1.000
	Community open space	-0.25	0.23	0.994
	Indoor marketplace	0.01	0.24	1.000
	Waterfront	-0.25	0.24	0.997

Change in Preference (3 of 3)

Space type	Comparison space type	Mean difference	Standard error	*p* value
Playground	Transport facility	-0.01	0.22	1.000
	Street	-0.29	0.22	0.973
	Square	-0.32	0.22	0.957
	Recreational space	0.25	0.22	0.994
	Found neighborhood space	0.29	0.23	0.983
	Park	0.03	0.22	1.000
	Memorial	1.07	0.22	0.000
	Market	-0.14	0.23	1.000
	Community open space	-0.39	0.22	0.809
	Indoor marketplace	-0.13	0.23	1.000
	Waterfront	-0.38	0.23	0.882
Community open space	Transport facility	0.38	0.22	0.837
	Street	0.10	0.21	1.000
	Square	0.07	0.22	1.000
	Recreational space	0.64	0.22	0.127
	Found neighborhood space	0.68	0.23	0.106
	Park	0.43	0.22	0.735
	Memorial	1.46	0.22	0.000
	Market	0.25	0.23	0.994
	Playground	0.39	0.22	0.809
	Indoor marketplace	0.26	0.23	0.992
	Waterfront	0.01	0.23	1.000
Indoor marketplace	Transport facility	0.12	0.23	1.000
	Street	-0.17	0.23	1.000
	Square	-0.19	0.24	1.000
	Recreational space	0.37	0.23	0.910
	Found neighborhood space	0.42	0.24	0.858
	Park	0.16	0.24	1.000
	Memorial	1.20	0.24	0.000
	Market	-0.01	0.24	1.000
	Playground	0.13	0.23	1.000
	Community open space	-0.26	0.23	0.992
	Waterfront	-0.26	0.24	0.996
Waterfront	Transport facility	0.37	0.23	0.902
	Street	0.09	0.23	1.000
	Square	0.06	0.23	1.000
	Recreational space	0.63	0.23	0.214
	Found neighborhood space	0.67	0.24	0.177
	Park	0.42	0.23	0.825
	Memorial	1.46	0.23	0.000
	Market	0.25	0.24	0.997
	Playground	0.38	0.23	0.882
	Community open space	-0.01	0.23	1.000
	Indoor marketplace	0.26	0.24	0.996
