# Supplementary material for: Effect of Street Performance (Busking) on the Environmental Perception of Public Space
Source: Front Psychol. 2021 Mar 30;12:647863. doi: 10.3389/fpsyg.2021.647863 (PMC8042224; doi:10.3389/fpsyg.2021.647863)
Supplement: Supplementary file 3 [file Data_Sheet_3.pdf]

Appendix C

Scale Items in Chinese

Visitability
如果我碰巧路過，我會在這個交匯點停留。
我會特意到訪並在這個交匯點消磨時間。
我會定期到訪這個交匯點。
這個交匯點是一個我會選擇在此與朋友見面的地方。
Restorativeness
在這個交匯點，我會能夠集中精神。
在這個交匯點，我會能夠專注於自己。
在這個交匯點，我會能夠放鬆。
在這個交匯點，我會能夠釋放所有壓力。
在這個交匯點，我的精力會得到恢復。
Preference
我十分喜歡這個交匯點。
我非常喜歡這個交匯點。
我會很享受這個交匯點。
我真的很享受這個交匯點。
